# Hepatitis C Prevalence, Incidence, and Treatment in Chinese Hemodialysis Patients: Results From the Dialysis Outcomes and Practice Patterns Study-China (2019–21)

**DOI:** 10.3389/fmed.2022.910840

**Published:** 2022-06-15

**Authors:** Liangying Gan, Dongyu Wang, Brian Bieber, Keith McCullough, Michel Jadoul, Ronald L. Pisoni, Fanfan Hou, Xinling Liang, Zhaohui Ni, Xiaonong Chen, Yuqing Chen, Li Zuo

**Affiliations:** ^1^Department of Nephrology, Peking University People's Hospital, Beijing, China; ^2^Arbor Research Collaborative for Health, Ann Arbor, MI, United States; ^3^Cliniques Universitaires Saint-Luc, Université Catholique de Louvain, Brussels, Belgium; ^4^State Key Laboratory of Organ Failure Research, Division of Nephrology, National Clinical Research Center of Kidney Disease, Nanfang Hospital, Southern Medical University, Guangzhou, China; ^5^Division of Nephrology, Guangdong Provincial People's Hospital, Guangdong Academy of Medical Sciences, Guangdong, China; ^6^Renal Division, Renji Hospital, Shanghai Jiao Tong University School of Medicine, Shanghai, China; ^7^Division of Nephrology, Ruijin Hospital, Shanghai Jiao Tong University School of Medicine, Shanghai, China; ^8^Renal Division, Peking University First Hospital, Beijing, China

**Keywords:** chronic kidney disease, hemodialysis, hepatitis C virus, prevalence, incidence, Dialysis Outcomes and Practice Patterns Study

## Abstract

**Background:**

Prior work from the Dialysis Outcomes and Practice Patterns Study (DOPPS) showed HCV prevalence in China in 2012–2015 being in the upper third and HCV incidence the 2nd highest among 15 different countries/regions investigated. The goal of the present investigation was to: (1) determine if HCV prevalence and incidence has changed, and (2) collect detailed data to understand how HCV is treated, monitored, and managed in Chinese HD facilities and non-dialysis chronic kidney disease (CKD) clinics.

**Data and Methods:**

Detailed data for 1,700 randomly selected HD patients were reported by 39 randomly selected HD facilities from Beijing, Shanghai, and Guangzhou participating in the DOPPS 7-China study from 2019 to 2021. The study site medical directors completed a survey regarding numerous aspects of HCV treatment and management in HD and ND-CKD patients.

**Results:**

In this 2019 to 2021 cohort, HCV prevalence was 7.4%, which was lower than the 14.8 and 11.5% HCV prevalence for the 2009–2011 and 2012–2015 cohorts, respectively. HCV incidence of 1.2 cases per 100 pt-yrs also was lower compared to the incidence of 2.1 for the 2012–2015 cohort. Although the great majority of study site medical directors indicated that all or nearly HCV+ patients should be treated for their HCV, very few HCV+ patients have been treated presumably due to substantial cost barriers for affording the new direct acting antivirals (DAAs). The randomly selected facilities in our DOPPS 7-China study appear to have excellent programs in place for frequent monitoring of patients and staff for HCV, education of staff, and referral of HCV cases to external infectious disease, gastroenterology, and liver disease specialists. Liver biopsies were not commonly performed in HCV+ HD patients. HCV genotyping also was rarely performed in participating units.

**Conclusions:**

Our study indicates a 50% decline in HCV prevalence and a >40% decline in HCV incidence in Chinese HD patients over the past 10–12 yrs. Chinese HD facilities and associated specialists appear to be well-equipped and organized for successfully treating and managing their HCV+ HD and CKD patients in order to achieve the WHO goal of eliminating HCV by 2030.

## Introduction

During the past 3 decades, patients receiving chronic dialysis as kidney replacement therapy for end stage kidney failure have been indicated to be at high risk for hepatitis C virus (HCV) infection. We have previously reported in the international, prospective Dialysis Outcomes and Practice Patterns Study (DOPPS) that HCV infection is common among in-center hemodialysis (HD) patients in >20 countries (1) and is associated with elevated all-cause mortality and morbidity, highly elevated rates of hepatic-related mortality and morbidity, and worse quality of life (2).

HCV has been reported to be the fourth-most commonly reported infectious disease in China, with ~10 million people infected (3). In addition, a disease sentinel surveillance program in China between 2010 and 2012, identified that the highest HCV seropositive rates in China were among persons using drugs and patients receiving hemodialysis (3). Previously, Jadoul et al. (1) reported an HCV prevalence of 11.5% among Chinese in-center chronic HD patients and an HCV incidence (95% CI) of 2.1(1.0, 4.2) new HCV cases per 100 patient-yrs based on a cohort of patients participating in DOPPS phase 5 data (2012–2015). The above HCV prevalence in China was in the upper third and HCV incidence the 2nd highest among the 15 different countries/regions investigated by Jadoul et al. (1).

However, the above HCV prevalence and incidence reported for China in DOPPS phase 5 was before direct acting antivirals (DAAs) were approved for treating HCV in China in 2017, and the management of blood-borne infectious diseases in hemodialysis facilities has been strengthened after establishing Hemodialysis Quality Control and Improvement Centers (HDQCIC) in each province. The current study was designed to describe the practice of HCV treatment among Chinese HD patients in phase 7 of the China DOPPS (2019–2021) and assess the impact of the availability of DAAs upon the trend in HCV prevalence and incidence.

## Methods and Materials

### Patients and Data Collection

Analyses were based on data collected in DOPPS 7-China as part of the 7th phase of the broader international Dialysis Outcomes and Practice Patterns Study (DOPPS). DOPPS is a prospective cohort study of HD patients ≥18 years of age. The present study included 1,700 Chinese HD patients, who participated in DOPPS 7 anytime between April 2019 and October 2021. Study participants were randomly selected for study participation from a representative sample of HD dialysis facilities in Beijing (*N* = 14 facilities), Shanghai (*N* = 13 facilities), and Guangzhou (*N* = 15 facilities) as described previously (4). All study sites were hospital-based with 56% being academic vs. 44% non-academic, and 55% designated as grade 3 hospitals vs. 45% as grade 2 hospitals. All Chinese study sites which participated in DOPPS 7 also had previously participated in DOPPS 5 (2012–2015), although there were 3 military hospital dialysis units that participated in China-DOPPS 5 which did not participate in DOPPS 7. The current investigation was based on patient- and facility-level data from 39 randomly selected Chinese HD facilities participating in DOPPS 7-China from April 2019 to October 2021 and which had completed a Medical Director's Survey on HCV Management and Monitoring. Study approval was obtained by a central institutional review board. Additional study approval and patient consent were obtained as required by national and local ethics committee regulations.

Patients from the initial prevalent cross-section of DOPPS 7 participants were included in analyses of overall HCV prevalence and comparison of patient characteristics by HCV status. From a total population of 1,700 study patients, the following exclusions were implemented (a) two facilities did not accept HCV+ patients (*n* = 69), (b) an additional 350 patients were not in the initial prevalent cross section, and (c) 2 patients were missing HCV status at enrollment for an overall sample of 1,279 patients used for prevalence analyses. When calculating HCV incidence among all patients the following exclusion criteria were applied: (a) two facilities did not accept HCV+ patients (*n* = 69), (b) 92 patients had an indication of HCV infection history at study enrollment, and (c) 342 patients had fewer than two HCV antibody tests during follow-up for an overall sample of 1,197 patients when calculating HCV incidence.

Demographic data, comorbid conditions, and laboratory values were abstracted from patient records. Baseline HCV status was determined based on an established diagnosis of HCV infection or positive HCV serology at DOPPS enrollment. HCV serology (quantitative and qualitative) were collected monthly. Information regarding each study site's treatment, management, screening and monitoring practices for HCV in their HD patients and non-dialysis chronic kidney disease (CKD) stages 3–5 patients was obtained by administering a specific HCV survey for each facility's Medical Director. These HCV surveys were completed by 39 of the 42 DOPPS 7-China study site Medical Directors, with responses used for analyses described in this investigation. Completion dates for these surveys were from December 2020 to September 2021, with 90% of the surveys completed between December 2020 and June 2021.

### Data Analysis

Seroconversion models and rates were restricted to patients with an initial negative HCV antibody measurement and at least one follow-up HCV antibody value. Time at risk started at the lab date for the first negative HCV antibody measurement (at study baseline or during follow-up) and ended at the seroconversion lab date or last negative HCV antibody lab date. Patients departing the study before a follow-up HCV antibody was measured were not included in the incident HCV seroconversion analyses. The median follow-up time for HCV seroconversion was 1.2 years.

Standard descriptive statistics were used to characterize the DOPPS patients included in the study as well as HCV prevalence, incidence, and facility HCV control practices. All analyses used SAS software, version 9.4 (SAS institute, Cary, NC).

## Results

### Study Sample Characteristics

Of the 39 facilities, 12, 12, and 15 of the facilities were from Beijing, Shanghai, and Guangzhou, respectively. Table 1 provides a comparison of the characteristics of HCV+ vs. HCV- patients from the initial cross-section of 1,279 Chinese HD patients in facilities participating in DOPPS 7. In this cross-section, 81 of the 1,279 patients were HCV+. This corresponds to 6.3% of patients but 7.4% when weighted by the facility sampling fraction. HCV+ patients had a lower mean age of 58.9 yrs (vs. 60.9 yrs among HCV- patients), and a much longer mean time on dialysis of 10.8 yrs (vs. 6.1 yrs among HCV- patients). HCV+ patients were almost evenly split between males and females whereas HCV- patients were more likely to be male. The prevalence of hepatitis B was 1.6 fold higher in HCV+ patients (8%) compared to HCV- patients (5%). However, the prevalence of coronary artery disease, hypertension, other cardiovascular disease, congestive heart failure, and cerebrovascular disease was lower among HCV+ vs. HCV- patients, whereas the prevalence of diabetes and peripheral vascular disease was very similar in HCV+ vs. HCV- patients. The prevalence of other displayed characteristics was quite low overall (<5%) with absolute differences consequently being relatively small in magnitude.

**Table 1:** Baseline patient characteristics of Chinese HD study patients, by HCV+ (China DOPPS, 2019–2021; initial cross-section of patients).

**Characteristics**	**HCV+**	**HCV-**	**Overall**
Number of patients	81	1,198	1,279
**Demographics**			
Age	58.9 (12.7)	60.9 (13.5)	60.7 (13.5)
Male Sex	51%	58%	58%
ESRD vintage, years	10.8 (8.1)	6.1 (4.9)	6.4 (5.3)
**Comorbidities**			
Hypertension	81%	87%	86%
Diabetes	33%	33%	33%
Coronary artery disease	20%	31%	30%
Cerebrovascular disease	10%	15%	15%
Peripheral vascular disease	11%	11%	11%
Congestive heart failure	22%	23%	23%
Cardiovascular disease, other	15%	19%	19%
Cancer	1%	4%	4%
Lung disease	5%	4%	4%
GI bleed in the last year	3%	2%	2%
Recurrent cellulitis	1%	2%	2%
Neurologic disorder	0%	6%	5%
Psychiatric disorder	3%	1%	1%
HIV/AIDS	0%	0%	0%
Hepatitis B	8%	5%	5%
Substance abuse in last 12 mo	0%	0%	0%
Cirrhosis	1%	1%	1%

### Prevalence and Incidence of HCV in Chinese HD Patients in DOPPS 7-China (2019–2021)

Although 6.3% of the initial cross-section of 1,229 study patients were HCV+, once analyses were weighted by the fraction of patients sampled within each HD facility, the prevalence of HCV+ was calculated to be 7.4% (Table 2). For comparison, we have shown that this HCV+ prevalence seen among Chinese HD patients in DOPPS 7 is lower than that which we reported previously in DOPPS for China. These new results from DOPPS 7 show a continual decline in HCV+ prevalence in Chinese HD patients from 14.8% in DOPPS 4 (2009–2011) to 11.5% in DOPPS 5 (2012–2015), and now to 7.4% in DOPPS 7 (2019–2021) despite 48.7% of HD facilities used the more sensitive PCR method in routine HCV screening (Table 3). DOPPS did not collect these data in China during the period from 2016 to 2018.

**Table 2:** HCV prevalence among Chinese HD Patients in DOPPS, at baseline by study phase.

**DOPPS Study Phase**	**Time frame**	**Prevalence**	***N* Pts**
4^*^	2009–2011	14.8%	1,223
5^*^	2012–2015	11.5%	967
7	2019–2021	7.4%	1,279

*Prevalence weighted by facility sampling fraction, excluding facilities that did not accept HCV+ patients*.

* *Published previously by Jadoul et al. (1)*.

**Table 3:** Method typically used for routine HCV screening in facility HD patients (China Medical Director HCV survey, 2021).

**HCV screening test**	**% of respondents (*n* = 39)**
HCV antibody test	51.3
HCV RNA test (PCR)	5.1
Both	43.6

Further analyses revealed an incidence rate of 1.2 new cases of HCV per 100 patient-years based on 16 new cases arising during follow-up of 1,197 patients who had data that met the analysis criteria introduced earlier (Table 4). This incidence rate is approximately half the rate of 2.1 new cases of HCV per 100 patient-years that we had determined previously for Chinese HD patients in DOPPS 5 (2012–2015). HCV antibody tests were reported as the method used for routine HCV screening by 51% of medical directors while 44% reported using both HCV antibody and HCV RNA (PCR) tests (Table 4). Although the number of incident HCV cases was too small for formal statistical analyses, several risk factors or HCV seroconversion were explored descriptively. The median (IQR) facility percent of patients with HCV at enrollment for units without any seroconverters (*n* = 28) vs. those with a seroconverter (*n* = 10) was 2.5% (0.0–4.8%) vs. 5.3% (2.5–7.9%). One seroconverter received a transfusion among 70 unique patients with a transfusion in China DOPPS 7. All incident HCV+ patients were HIV negative and had no substance abuse history in the last 12 months. The percentage of hepatitis B positivity in incident HCV+ patients vs. HCV- patients was 6.3 vs. 4.8%.

**Table 4:** Incidence of becoming HCV+ during study follow-up, among Chinese HD Patients in DOPPS, by study phase.

**DOPPS study phase**	**Time frame**	**HCV+ Incidence (per 100 patient-years)**	***N* Cases**	**N patients**
4^*^	2009–2011	–		
5^¶^	2012–2015	2.1	8	450
7	2019–2021	1.2	16	1,197

* *Not determined in phase 4 data because HCV antibodies were not collected longitudinally in China DOPPS Study Phase 4*.

¶ *Published previously by Jadoul et al. (1). Restricted to patients with at least two HCV antibody measurements and in whom the initial HCV antibody measurement was negative, excluding facilities that did not accept HCV+ patients*.

### HCV Treatment in DOPPS 7-China HD Patients During Study Follow-Up

Among the 97 patients noted to be HCV+ at study baseline or who became HCV+ during study follow-up, anti-HCV medications were prescribed for 9 of these patients (Table 5): 4 of 9 patients (44%) were prescribed Epclusa (sofosbuvir + velpatasvir), 1 each prescribed Daklinza (daclatasvir), Interferon beta, and Sovaldi (sofosbuvir), and with no drug name reported for 2 treated patients.

**Table 5:** Treatment of HCV+ patients based on patient-level prescription data (China DOPPS, 2019–2021).

**Medication**	***N* Patients**
Epclusa (sofosbuvir + velpatasvir)	4
Daklinza (daclatasvir)	1
Interferon beta	1
Sovaldi (sofosbuvir)	1
Other	2

The type of HCV medication used to treat HCV+ dialysis patients was also indicated by 13 of the 39 China-DOPPS 7 medical directors (Table 6), showing greater diversity in the range of medications used for treating HCV compared to the HCV medications prescribed for the relatively small number of 9 patients treated during follow-up as described above. At 4 of these 13 HD units, HCV was typically treated with interferon alone or with ribavirin; 3 of the 13 HD units used only Zepatier, and 1 site used only Epclusa, 1 used Epclusa or Zepatier, 1 used Epclusa or Viekira Pak, 1 used Epclusa, interferon or ribavirin, 1 used Epclusa, Harvoni, Ribavirin or Interferon, and 1 used either Epclusa, Harvoni, Sovaldi, Vosevi, Viekira Pak, or interferon + ribavirin; 2 of the 39 facilities indicated they did not accept HCV+ patients, 3 facilities indicated that they didn't currently have any HCV+ patients (and didn't answer the medication question), and 21 facilities (53%) indicated that HCV treatment was administered outside of their facility.

**Table 6:** Medical director responses regarding the types of medications used by DOPPS 7-China HD units for treating HCV+ in their HD patients (China Medical Director HCV survey, 2021).

**Medication (s)**							**Frequency (*n* = 39)**
Interferon and/or ribavirin							4
Interferon and/or ribavirin	Epclusa						1
Interferon and/or ribavirin		Harvoni					1
Interferon and/or ribavirin	Epclusa	Harvoni	Sovalid	Viekira Pack	Vosevi		1
	Epclusa						1
						Zepatier	3
	Epclusa					Zepatier	1
	Epclusa			Viekira Pack			1
HCV+ patients not accepted at facility							2
Not currently any HCV+ patients at facility							3
HCV treatment not administered at facility							21

### Medical Director Survey Responses on HCV Management and Monitoring

#### Perspectives on Treating HCV

Surveys regarding the treatment, management and monitoring of HCV were completed by the medical directors from 39 of the 42 DOPPS 7-China study sites. Nearly all medical directors (92 and 95%) indicated that all or nearly all HCV+ dialysis patients on a kidney transplant waiting list or HCV+ CKD stage 3 patients should be treated with antiviral medication for HCV (Table 7), and with a similar, although slightly lower percentage for the treatment of HCV+ dialysis patients NOT on a kidney transplant waiting list or having CKD stage 4–5 patients. In contrast to the above perspectives, when asked what % of current HCV+ patients had ever been treated with anti-HCV medications at any time, by any physicians managing HCV care (Table 8), ~50% of medical directors indicated that none of their current HCV+ dialysis patients had ever been treated with an anti-HCV medication, whereas ~20% of medical directors indicated that >50% of current HCV+ dialysis patients had ever been treated with an anti-HCV medication. Similar results were noted in HCV treatment of CKD stage 3–5 HCV+ patients.

**Table 7:** Medical director perspectives of whether HCV+ dialysis or CKD patients should be treated with an anti-viral medication (China Medical Director HCV survey, 2021).

	**HD-tx waitlisted (% of *n* = 38)**	**HD-not tx waitlisted (% of *n* = 39)**	**CKD stage 3 (% of *n* = 39)**	**CKD stage 4 (% of *n* = 38)**
Agree	92.1	84.6	94.9	86.8
Neutral	7.9	15.4	5.1	13.2
Disagree	0.0	0.0	0.0	0.0

**Table 8:** Estimated % of clinic HCV+ patients that have been treated with anti-HCV medications, by ESKD stage (China Medical Director HCV survey, 2021).

	**HD-tx waitlisted**	**HD-not tx waitlisted**	**CKD stage 3**	**CKD stage 4**
**% of patients treated**	**(% of *n* = 39)**	**(% of *n* = 39)**	**(% of *n* = 38)**	**(% of *n* = 39)**
0%	56.4	46.2	44.7	48.7
1–5%	10.3	20.5	18.4	15.4
6–10%	0.0	5.1	7.9	7.7
11–25%	0.0	2.6	2.6	2.6
26–50%	10.3	7.7	2.6	5.1
>50%	23.1	17.9	23.7	20.5

#### Type of Doctor Who Typically Treats HCV in Dialysis or ND-CKD Patients

When dialysis unit medical directors were asked to what degree they felt comfortable or adequately trained in treating HCV in their HCV+ patients, 15% of medical directors indicated being somewhat or very comfortable, 44% were neutral, and 41% felt somewhat or very uncomfortable in treating HCV in their HCV+ patients (Table 9). Consequently, at the majority of DOPPS 7-China HD units, the type of doctor who usually prescribes antiviral medications to treat HCV in their dialysis patients typically is not a nephrologist with: 40% of study sites indicating this to be an infectious disease specialist, 30% indicating a liver or gastroenterology specialist, only 11% indicating a nephrologist, 8% indicating a type of doctor different than those above, and 11% of sites indicating that their facility's practice is to NOT have hepatitis C treated in their HCV+ patients (Table 10A). Furthermore, 85% of medical directors indicated that their HCV+ dialysis patients typically were referred to a liver or gastroenterology specialist as part of their care (Table 10B). Medical director responses were nearly identical for HCV treatment of non-dialysis CKD stage 3–5 patients vs. dialysis patients with regard to the above two questions.

**Table 9:** Do you feel comfortable/adequately trained in treating HCV in your facility's HCV+ patients? (China Medical Director HCV survey, 2021).

**Comfort level treating HCV**	**% of respondents (*n* = 39)**
Very comfortable	5.1
Somewhat comfortable	10.3
Neutral	43.6
Somewhat uncomfortable	23.1
Very uncomfortable	17.9

**Table 10A:** Physician type who usually prescribed antiviral medications to treat HCV+ patients, by ESKD stage (China Medical Director HCV survey, 2021).

	**HD**	**CKD stage 3**	**CKD stage 4**
**HCV medication prescriber**	**(% of *n* = 38)**	**(% of *n* = 38)**	**(% of *n* = 37)**
Nephrologist	10.5	10.5	10.8
Liver or gastroenterology specialist	28.9	28.9	29.7
Primary care physician	0.0	0.0	0.0
Infectious disease specialist	42.1	42.1	43.2
Other	7.9	7.9	8.1
HCV+ patients not treated	10.5	10.5	8.1

**Table 10B:** HCV+ patients are usually referred to a liver/gastroenterology specialist, by ESKD stage (China Medical Director HCV survey, 2021).

	**HD**	**CKD stage 3**	**CKD stage 4**
**Referred to specialist**	**(% of *n* = 39)**	**(% of *n* = 39)**	**(% of *n* = 39)**
Yes	84.6	87.2	87.2
No	15.4	12.8	12.8

#### Testing for HCV Genotypes and Frequency of Liver Biopsies

In asking about the percent of a HD facility's HCV+ patients for whom HCV genotyping was performed either for new HCV cases or when the HCV genotype is unknown, 85% of unit medical directors indicated that HCV genotyping is performed only for 1–20% of such HCV+ patients in their dialysis units, with 6 and 9% of medical directors, respectively, indicating that HCV genotyping was performed for 41–80% and 81–100% of their HCV+ HD patients (Table 11). Only a small fraction of unit medical directors–13 and 5%, respectively, —were aware of whether their HD unit's HCV genotype 1 patients were tested for NS5A resistance polymorphisms or whether their HCV genotype 1a dialysis patients were tested for the NS3 Q80K mutation (Table 11). Medical directors were also questioned about what % of HCV+ patients had ever had a liver biopsy (Table 12). Medical director responses indicated that no liver biopsies had been performed for any of their current HCV+ patients in ~70% of HD units and 72% of CKD clinics treating CKD stage 3 and 4 patients. Medical directors indicate that only 3–5% of HD facilities and 3–5% of ND-CKD clinics had performed a liver biopsy in >50% of their HCV+ patients.

**Table 11:** HCV genotyping practices (China Medical Director HCV survey, 2021).

**Genotyping practice**	**% of responses (*n* = 39)**
**% HCV+ patients genotyped** ^**a**^	
1–20%	84.6
21–40%	2.6
41–60%	2.6
61–80%	2.6
81–100%	7.7
**Genotyping awareness**	
NS5A resistance associated polymorphisms^b^	12.8
NS3 Q80K mutation^c^	5.1

a *Either for new HCV cases or when the HCV genotype is unknown*.

b *Are you typically aware whether your HD facility's HCV genotype 1 dialysis patients are tested for NS5A resistance associated polymorphisms (e.g., amino acid substitutions at positions 28, 30, 31, 58, 93)?*

c *Are you typically aware whether your HD facility's HCV genotype 1a dialysis patients are tested for the NS3 Q80K mutation?*

**Table 12:** Estimated percent of facility HCV+ patients who have ever had a liver biopsy, by ESKD stage (China Medical Director HCV survey, 2021).

**% of patients with biopsy**	**HD-tx waitlisted (% of *n* = 39)**	**HD-not tx waitlisted (% of *n* = 38)**	**CKD stage 3 (% of *n* = 39)**	**CKD stage 4 (% of *n* = 39)**
0%	79.5	73.7	69.2	71.8
1–5%	2.6	10.5	12.8	10.3
6–10%	2.6	2.6	5.1	5.1
11–25%	0.0	2.6	2.6	2.6
26–50%	12.8	5.3	5.1	7.7
>50%	2.6	5.3	5.1	2.6

#### Screening for HCV in HD Facility or in ND-CKD Patients

Medical directors were asked the frequency at which HD patients in their facility were typically screened for HCV (Table 13A), with 92% indicating twice per year or more often, 6% indicating once per year or less often, and 3% only when a patient joins their facility. However, compared to HCV screening in dialysis patients, the frequency of HCV screening of non-dialysis CKD stage 3 or 4 patients at many facilities is considerably less frequent, with ~50% indicating twice per year or more often, 28% indicating only once per year or less often than once per year, and ~22% screening for HCV only when a patient joins their CKD clinic. A difference was seen as well in practices for monitoring HCV trends in dialysis vs. non-dialysis CKD stage 3–5 patients (Table 13B): 31, 54, and 5% of medical directors indicated that their facilities monitor HCV trends in their dialysis patients on a monthly, twice per year, or once per year frequency, respectively; by contrast, 62, 12, and 20% of medical directors indicate that their facilities monitor HCV trends in their ND-CKD patients on a monthly, twice per year, or once per year frequency, respectively. Finally, nearly all HD unit medical directors (95–97%) indicated that their HD patients are typically screened for HCV after returning from travel or a long-term hospitalization (Table 13C).

**Table 13A:** Frequency of screening for HCV, by ESKD stage (China Medical Director HCV survey, 2021).

**HCV screening**	**HD**	**CKD stage 3**	**CKD stage 4**
**frequency**	**(% of *n* = 39)**	**(% of *n* = 39)**	**(% of *n* = 39)**
At least every 3 months	25.6	33.3	33.3
Twice per year	66.7	17.9	15.4
Once per year	2.6	20.5	20.5
Less than once per year	2.6	7.7	7.7
Only upon entry to our facility	2.6	20.5	23.1

**Table 13B:** Frequency of monitoring facility HCV trends, by ESKD stage (China Medical Director HCV survey, 2021).

**Facility HCV trend**	**HD**	**CKD stage 3**	**CKD stage 4**
**monitoring frequency**	**(% of *n* = 39)**	**(% of *n* = 39)**	**(% of *n* = 39)**
At least monthly	30.8	61.5	59
~3 times per year	10.3	2.6	5.1
~2 times per year	53.8	12.8	10.3
~1 time per year	5.1	17.9	20.5
Less than 1 time per year	0.0	5.1	5.1

**Table 13C:** Situations after which HCV testing is typically performed (China Medical Director HCV survey, 2021).

**Situation**	**Yes (%)**	**No (%)**	**Not applicable (%)**	**Responses (n)**
Travel to a country known to have high HCV prevalence	97.4	0.0	2.6	39
Return to facility after long-term hospitalization	94.9	5.1	–	39

#### Dialysis Unit Staff HCV Education and HCV Screening

Medical directors indicated that screening of dialysis unit staff members for HCV was very common across nearly all China-DOPPS HD study sites with most sites (92%) screening staff members once per year, 5% screening twice per year, and 3% screening less often than once per year (Table 14). In addition, 95% of dialysis units provide staff members with educational courses on prevention of transmission of blood borne viruses (including HCV) once per year, 3% once every 2 years, and 3% less often than every 2 years (Table 14). In addition, 97% of dialysis units instruct new staff members about HCV precautions as part of their orientation.

**Table 14:** HCV education and screening of dialysis staff members (China Medical Director HCV survey, 2021).

**Practice**	**Prevalence (%)**
**Staff HCV screening frequency** ^**a**^	
At least 2 times per year	5.1
~1 time per year	92.3
Less often than 1 time per year	2.6
**Staff HCV training frequency** ^**b**^	
1 time per year	94.9
One time every 2 years	2.6
Less often than every 2 years	2.6
**Staff orientation includes HCV precaution instructions** ^**c**^	
Yes	97.4
No	2.6

a *N = 39 responses*.

b *n = 39 responses; How often does your dialysis unit provide staff members with educational courses on prevention of transmission of blood borne viruses (including HCV)?*

c *n = 38 responses; Does your dialysis unit instruct new staff members about HCV precautions as part of their orientation?*

## Discussion

In China, the main high-risk populations for HCV infection have been reported to be intravenous drug users, patients receiving hemodialysis, and patients co-infected with HIV or hepatitis B (5). In view of the large population of individuals receiving hemodialysis for end stage kidney failure in China, great efforts have been underway for many years to reduce HCV infections in this patient population. The present study represents the most detailed investigation of the prevalence, incidence, treatment, and monitoring of HCV in chronic HD patients in China since direct acting antiviral medications were approved for HCV treatment in China in 2017.

Our study results from the DOPPS 7-China indicate a 50% decline in HCV+ prevalence in Chinese HD patients over the past 10–12 yrs, with a prevalence of 7.4% seen in 2019–2021 compared to 11.5% in DOPPS 5 (2012–2015) and 14.8% in DOPPS 4 (2009–2011) (1). The above possible substantial decline in HCV prevalence within the DOPPS-China study sites over time has been seen despite the possibility that detection of HCV infected HD patients may have increased due to PCR more commonly being used as part of HCV monitoring activities in more recent years. Similar to what we had seen previously in an analysis of HCV across 15 countries/international regions in DOPPS (1), HCV+ HD patients in China were more likely to be younger, had a considerably longer mean number of years on dialysis, and were much more likely to also be hepatitis B+. However, the percentage of HD patients who were female was higher among HCV+ vs. HCV- patients in China in contrast to our prior international findings in which the proportion of female patients was lower among HCV+ individuals. The lower prevalence of cardiovascular disease comorbidities seen among HCV+ patients in our Chinese study sample may be a reflection in part of the younger mean age of HCV+ patients.

Consistent with the above trend in declining HCV prevalence among Chinese HD patients, our analyses also indicate an HCV incidence of 1.2 new cases of HCV per 100 patient-yrs seen among study patients who became HCV antibody positive during study follow-up in DOPPS 7-China (2019–2021). This incidence rate is >40% lower than the rate of 2.1 new cases of HCV per 100 patient-yrs that Jadoul et al. (1) had reported previously for Chinese HD patients in DOPPS 5 (2012–2015).

The large improvement seen in the incidence and prevalence of HCV in HD patients in China-DOPPS could be a reflection of the extensive HCV management program in place at many of the DOPPS 7-China study sites under the direction of local HDQCIC which was first established in Shanghai in 1999, followed by Beijing in 2002 (6). Strict adherence to infection control procedures and routine serologic screening plays a pivotal role in preventing transmission of HCV within hemodialysis units (7, 8). HDQCIC pushes hemodialysis facilities to formulate infection control procedures including routine training, HCV screening for new HD patients or patients transferring from another hemodialysis facility followed by retesting every 6–12 months for all HCV- hemodialysis patients, and implementation of optimal hygienic precautions. Also HDQCIC does routine observational audits of various infection control practices combined with feedback of results to clinical staff in accordance with KDIGO hepatitis C guidelines (8).

This well-developed infection control monitoring program is reflected by nearly all medical directors in DOPPS China facilities reporting frequent monitoring for HCV, with 26 and 67% of HD units screening their patients for HCV every 3 or 6 months, respectively. Additionally, 31% of HD units monitor their trends in HCV positivity every month, while 64% monitor their HCV+ trends 2–3 times per yr. Moreover, in ~95% of facilities, patients are typically tested for HCV when returning to the facility after a long-term hospitalization or having traveled to a country known to have high HCV prevalence. Regular HCV testing is also applied to staff, with 97% of DOPPS 7-China HD units screening dialysis unit staff at least once per year for HCV. Furthermore, 95% of HD units have a HCV training course for staff each year, and orientation of new staff includes HCV precaution instructions at 97% of HD units.

DAAs were approved for treating HCV in China in 2017 which is later than some other developed countries and the safety and efficacy of DAA therapy in CKD have been demonstrated by a number of studies (9–11). From the medical directors survey we get the answer about whether application of DAAs in HD patients is a reason for the declining HCV prevalence and incidence in Chinese HD patients. Although nearly all China DOPPS medical directors (92+%) indicated that their HCV+ should be treated with DAAs, ~50% of medical directors indicated that none of their current HCV+ dialysis patients had ever been treated with an anti-HCV medication (consistent with patient-level data). Reasons are likely multifactorial for the low level of antiviral treatment of HCV+ patients in practice among the DOPPS 7-China HD units, but we suspect the main factor is the high cost of DAAs accompanied by lack of reimbursement by the national insurance system during this study time period (12). A similar situation occurred in Taiwan, where DAAs were approved for use in 2015, but the number of patients receiving DAAs treatment was very limited until 2018 due to medical insurance reimbursement policy (13). In our study, HCV+ patients were referred to a liver/gastroenterology specialist in 85% of HD units. Furthermore, in most facilities, prescription of antiviral medications to treat HCV+ patients was typically managed by an infectious disease or liver/gastroenterology specialist, with nephrologists prescribing antiviral medications for their HCV+ patients in only 11% of HD units. So adequate access to DAAs in dialysis patients in China also depends on good collaboration between nephrologists and other specialists (14).

When DAAs were used, Medical Director responses and the few patient-level prescriptions demonstrate that a broad array of different DAAs are being used across these study sites—and in different combinations—indicating substantial diversity in how DAAs are being applied in different HD units. This is partly due to the fact that these drugs were not available at the same time in China, and the drug choices available in different hospitals were not equal. Daclatasvir was one of the earliest DAAs available in China, while DAAs such as Pibrentasvir/Glecaprevir or Elbasvir/Grazoprevir, which are more suitable for patients with end-stage renal disease, entered China much later.

Although HCV was not treated in many HCV+ HD patients as discussed above, Medical Director responses provide a clear picture that management and monitoring of HCV appeared to be well-organized across the vast majority of study site HD units. After 2020, Elbasvir/Grazoprevir, Ledipasvir/Sofosbuvir, and Sofosbuvir/Velpatasvir were successfully reimbursed by the national insurance system, with an average price drop of more than 85% (15). These drugs are available gradually in different hospitals and the costs of these drugs are affordable after reimbursement now. Physicians, nephrologists, or other specialists will be free to choose DAAs suitable for dialysis patients. With proper quality improvement initiatives, HCV elimination from dialysis facilities is possible (16, 17).

Since 7.4% is still a relatively high HCV prevalence, the nosocomial transmission signs in China DOPPS7 should also be explored. One of the 16 incident HCV+ patients had history of a blood transfusion during DOPPS follow-up. Transfusion safety has been greatly improved in China with more sensitive nucleic acid testing (NAT) screening for HCV being widely used in blood donors (1). However, the HCV incidence in repeat blood donors in China was 15.2 per 100,000 person-years which indicates that there remains some infection risk because of the time window/period of donation and thus HCV infection is still an important concern for transfusion safety (18). As previously published in DOPPS (1), facility HCV prevalence is higher in facilities with seroconverters than in facilities without seroconverters (5.3 vs. 2.5%). Facility HCV prevalence is also a risk factor of seroconversion in China. As suggested by KDIGO hepatitis guideline, strategies to prevent HCV transmission within hemodialysis units should prioritize adherence to standard infection control practices (8).

Although a strength of the DOPPS 7–China study is the random selection of HD units for participation in the study, stratified to represent both tier 2 and 3 hospitals, a limitation of our study is that it does not represent all Chinese dialysis/CKD practice in being limited to hospital-based facilities located in the metropolitan areas of Beijing, Shanghai, and Guangzhou, and HCV prevalence is known to vary considerably across different regions of China (19, 20). However, the current study facility sample provides the ability to compare with earlier DOPPS-China results of HCV prevalence and incidence which included many of the same facilities as those participating in DOPPS 7-China. Additionally, the methodology and surveys used in the present study hopefully can serve as the basis for broader capture of these numerous aspects of HCV care in HD units in other Chinese jurisdictions to more thoroughly understand the variability in HD patient HCV incidence, prevalence and treatment across Chinese HD facilities. Another limitation of our study is that since HCV treatment for most HD patients is managed by non-nephrologist specialists, it is conceivable that HCV genotyping and anti-HCV medications prescribed for HCV+ dialysis patients were not always captured within a dialysis unit's medical record for such care provided outside of the dialysis unit. Additionally, even though PCR HCV viral load data were reported for >500 viral load measurements for study patients during follow-up, the diversity in how these values were reported for patients by different HD study sites, and in the common occurrence of duplicate values being reported over a 3 month period made interpretation challenging. This latter experience suggests the need for standardized measurement and reporting systems for PCR HCV viral load levels to be implemented across Chinese HD units if desiring to capture viral load data in a meaningful and interpretable fashion across HD facilities as one aspect of a broad-based HCV surveillance system for China.

In conclusion, this latest investigation of HCV in Chinese HD patients as viewed through the lens of the DOPPS-China study in Beijing, Shanghai, and Guangzhou documents a decline in HCV prevalence and incidence in Chinese HD patients when compared to earlier DOPPS-China results. The randomly selected HD units in the DOPPS 7-China study appear to have excellent programs in place for frequent monitoring of patients and staff for HCV, education of staff, and referral of HCV cases in most HD units to external infectious disease, gastroenterology, and liver disease specialists. Even though the great majority of study site medical directors feel that all or nearly HCV+ patients should be treated, very few if any HCV+ patients have been treated presumably due to substantial cost barriers for affording the new DAAs. DAAs are now covered by the Chinese National Medical Insurance reimbursement system which will likely lead to widespread use of DAAs and further reductions in the incidence and prevalence of HCV in this large patient population.

## Data Availability Statement

Data may be made available to qualified researchers for approved scientific uses. Some limitations and fees may apply. Arbor Research Collaborative for Health encourages investigators, whether or not previously affiliated with the Dialysis Outcomes and Practice Patterns Study (DOPPS), to submit proposals for data use, collaboration, and ancillary studies.

## Ethics Statement

The studies involving human participants were reviewed and approved by the Ethics Committee of Peking University People's Hospital (ethical approval number: 2018PHB028-01). The patients/participants provided their written informed consent to participate in this study.

## Author Contributions

All authors listed have made a substantial, direct, and intellectual contribution to the work and approved it for publication.

## Funding

This work has been supported in part by a research grant from Investigator-Initiated Studies Program of Merck Sharp & Dohme Corp. Global support for the ongoing DOPPS Programs is provided without restriction on publications by a variety of funders. For details, see https://www.dopps.org/AboutUs/Support.aspx. All grants were made to Arbor Research Collaborative for Health and not to coauthors directly.

## Author Disclaimer

The opinions expressed in this paper are those of the authors and do not necessarily represent those of Merck Sharp & Dohme Corp.

## Conflict of Interest

MJ has been a speaker or consultant for Abbvie and Merck and has received an unrestricted educational grant from Merck. The remaining authors declare that the research was conducted in the absence of any commercial or financial relationships that could be construed as a potential conflict of interest.

## Publisher's Note

All claims expressed in this article are solely those of the authors and do not necessarily represent those of their affiliated organizations, or those of the publisher, the editors and the reviewers. Any product that may be evaluated in this article, or claim that may be made by its manufacturer, is not guaranteed or endorsed by the publisher.
